# Mobile health assisted self-monitoring is acceptable for supporting weight loss in rural men: a pragmatic randomized controlled feasibility trial

**DOI:** 10.1186/s12889-021-11618-7

**Published:** 2021-08-18

**Authors:** Christine M. Eisenhauer, Fabiana Brito, Kevin Kupzyk, Aaron Yoder, Fabio Almeida, Rebecca Johnson Beller, Jessica Miller, Patricia A. Hageman

**Affiliations:** 1 College of Nursing-Northern Division, University of Nebraska Medical Center, 801 East Benjamin Avenue, Norfolk, NE 68701 USA; 2 College of Public Health, University of Nebraska Medical Center, 984355 Medical Center, Omaha, NE 68198-4355 USA; 3 College of Nursing-Omaha Division, University of Nebraska Medical Center, 985330 Nebraska Medical Center, Omaha, NE 68198-5330 USA; 4 Department of Environmental, Agricultural & Occupational Health, College of Public Health, University of Nebraska Medical Center, 984388 Nebraska Medical Center, Omaha, NE 68198-4388 USA; 5 Department of Health Promotion, College of Public Health, University of Nebraska Medical Center, 984365 Nebraska Medical Center, Omaha, NE 68198-4365 USA; 6 Department of Health and Rehabilitation Sciences, College of Allied Health Professions, University of Nebraska Medical Center, 984420 Nebraska Medical Center, Omaha, NE 68198-4420 USA

**Keywords:** Rural population, Mobile health technologies, Men, Weight loss, Health disparities, Self-monitoring

## Abstract

**Background:**

Addressing overweight and obesity among men at-risk for obesity-related diseases and disability in rural communities is a public health issue. Commercial smartphone applications (apps) that promote self-monitoring for weight loss are widely available. Evidence is lacking regarding what support is required to enhance user engagement with and effectiveness of those technologies. Pragmatically comparing these apps effectiveness, including rural men’s desired forms of support when using them, can lead to greater weight loss intervention impact and reach. This study assessed the feasibility and acceptability of a mobile technology application applied differently across two groups for weight loss.

**Methods:**

In a two-armed, pragmatic pilot feasibility study, 80 overweight and obese men aged 40–69 were randomized using a 1:1 ratio to either an enhanced Mobile Technology Plus (MT+) intervention or a basic Mobile Technology (MT) intervention. The MT+ group had an enhanced smartphone app for self-monitoring (text messaging, discussion group, Wi-Fi scale) whereas the MT group received a basic app that allowed self-monitoring logging only. Assessments were collected at baseline, 3 and 6 months. App logs were analyzed to track engagement and adherence to self-monitoring. Acceptability was assessed via focus groups. Analysis included descriptive statistics and qualitative content analysis.

**Results:**

Of 80 men recruited, forty were allocated to each arm. All were included in the primary analysis. Recruitment ended after 10 months with a 97.5 and 92.5% (3 month, 6 month) retention rate. Over 90% of men reported via survey and focus groups that Lose-It app and smart scale (MT+) was an acceptable way to self-monitor weight, dietary intake and physical activity. Adherence to daily app self-monitoring of at least 800 dietary calories or more (reported respectively as MT+, MT) was positive with 73.4, 51.6% tracking at least 5 days a week. Adherence to tracking activity via recorded steps four or more days weekly was positive, 87.8, 64.6%. Men also adhered to self-weighing at least once weekly, 64, 46.3%. At 6 months, an observed mean weight loss was 7.03 kg (95% CI: 3.67, 10.39) for MT+ group and 4.14 kg (95% CI: 2.22, 6.06) for MT group, with 42.9 and 34.2% meeting ≥5% weight loss, respectively. No adverse events were reported.

**Conclusions:**

This National Institutes of Health-funded pilot study using mobile technologies to support behavior change for weight loss was found to be feasible and acceptable among midlife and older rural men. The interventions demonstrated successful reductions in weight, noting differing adherence to lifestyle behaviors of eating, monitoring and activity between groups, with men in the MT+ having more favorable results. These findings will be used to inform the design of a larger scale, clinical trial.

**Trial registration:**

The trial was prospectively registered with ClinicalTrials NCT03329079. 11/1/2017.

## Background

Obesogenic behaviors are a public health problem in the United States of America (USA) evidenced by a staggering 55 million men who are overweight or obese [1]. Rates of obesity have tripled in the last 20 years for men living in the rural Midwestern US states [2, 3]. As occupations in rural areas have evolved from human dependent industry jobs to employment that is technologically driven, there is a marked increase in sedentary work time, potentiating the risk for developing overweight/obesity in rural men [4, 5]. Globally, rural men have higher rates of overweight and obesity than urban men, with 55% of the rise in body mass indices recorded over the past three decades occurring in rural locales [6]. Overweight or obesity puts rural men at higher risk for developing diabetes, cardiovascular disease, and some cancers and overall worse health outcomes than urban men [7, 8].

Self-monitoring of physical activity, weight, and dietary intake is positively associated with weight loss [9]. Smartphone applications (apps) and other wearable technologies have demonstrated weight loss effectiveness in adults and in historically underserved and minority United States populations [10]. Few of these mHealth interventions, however, have involved tailoring or optimization [11]. Self-monitoring apps permit evaluation of one’s progress towards goals and facilitate real-time self-monitoring of health behaviors in an easy-to-use, accessible format [12–14]. Rural men are underrepresented in behavioral weight loss trials, with documented poor access to sources of preventive care and behavioral health counseling [15, 16]. Current reports estimate that 80% of rural men own a smartphone [17]. Within rural settings, the acceptability and feasibility of mobile health technologies for self-monitoring for weight loss is a largely uncharted area. Determining what technology-based approach to weight loss is most acceptable to rural men is key to selecting intervention components are most efficacious for this group in future trials [18].

Rural men are less likely to participate in traditional, face-to-face weight loss programs or self-monitoring of eating and activity than urban men [19–22]. Midwestern rural USA cultural norms reinforce the importance of stoicism, self-reliance, and masculinity in men [23], which may contribute to their avoidance of help-seeking behaviors [24]. Limited access to weight loss resources may be one reason [25].

There is evidence to support that tailored [26], app-based technologies support improvement in behavior performance, adherence, and motivation [27]. A gap remains in understanding what specific tailored supports are required to enhance user engagement with and the effectiveness of app-based weight loss technologies among rural men [28]. Examining the acceptability and feasibility of a commercially available app in different formats: one with enhanced self-monitoring and engagement support and the other the basic app version only, may improve our understanding of rural men’s uptake and preferences for use of these technologies [29, 30]. A feasible and acceptable weight loss intervention is needed that can attract and engage rural men for sustained self-monitoring (eating, physical activity, and self-weighing) behaviors, which may have a significant impact in addressing multiple obesity-related rural health disparities.

Accordingly, the aim of this study was to determine the feasibility and acceptability of an enhanced mobile technology delivered self-monitoring intervention (Mobile Technology Plus (MT+) for achieving weight loss in rural men. This feasibility study also proposed to establish point estimate and variability of outcome measures of weight loss (primary) and dietary and physical activity (secondary) at 3 and 6 months across two mobile technology interventions, MT+ and mobile technology basic (MT). We provide descriptive evaluation of this study’s feasibility and acceptability and discuss the preliminary outcome trends as aligned with current recommendations for reporting feasibility studies [31, 32].

## Methods

### Design

A two-arm, randomized controlled trial was conducted to determine the feasibility and acceptability of mobile health technologies for self-monitoring eating, activity, and weight for weight loss in rural men. A detailed description of the study protocol was published elsewhere [33] with a brief summary of the methods provided below. The study received ethics approval from the University of Nebraska Medical Center (UNMC) Institutional Review Board IRB#594–17-EP. All methods were performed in accordance with the Declaration of Helsinki and relevant guidelines and regulations. All study participants provided written informed consent.

### Study population

Eligible participants were male adults, aged 40–69 years, who reside in rural-designated Rural Urban Commuting Area (RUCA) code areas [34] of northeast Nebraska, USA, and who were overweight or obese with a body mass index (BMI) of 28 kg/m^2^ or higher and weight not > 396 pounds (due to weight limitation of smart scale). Inclusion criteria included owning a smartphone with enabled short message service (text); having an email account; answering “no” to all questions on the physical activity readiness questionnaire screener (PAR-Q17) [35, 36] or obtaining clinician clearance prior to enrollment; and be willing to share their app self-monitoring logs with the investigative team. Men were excluded from participating if they had experienced a recent weight loss of 5% or more in the past 6 months; currently taking medications that influenced weight; used the Lose-It! self-monitoring app in the past to lose weight; had a person from the same household enrolled in this study; or were type I diabetic or type II diabetic with insulin dependence.

### Recruitment

This study utilized several community engagement strategies previously deemed useful in this region to have demonstrated positive recruitment and retention: a community advisory board, and outreach to local leaders and area businesses, churches, and schools [24, 37]. Rural health professions students were also involved in outreach activities across aspects of study planning, implementation, and evaluation as a requirement of the funder’s goal to expose under-represented rural students to research. Recruitment of advisory board members, intervention participants, and focus group participants occurred during June 2018 to November 2019. Participant’s recruitment channels included: clinicaltrials.gov website, Facebook blasts, newspaper email list-serves, radio ads, community fairs, and print brochures. Inquiring men were screened for eligibility by telephone and informed consent was collected in real-time via HIPAA compliant REDCap data capture on their computer or smartphone. An in-depth study protocol describing these procedures has previously been published [33].

### Randomization

Eligible men were randomized in a 1:1 ratio in blocks of eight to accommodate a sufficient discussion cohort for the MT+ group. Randomization followed a statistician-derived allocation schedule that used a random number generator. Due to the nature of the study, participants and outcome assessor (a public health worker) were aware of intervention allocation. Though the assessor had no role in the intervention delivery, he was notified of each participant’s REDCap code for survey access and entry, participant-specific username and password for the app, and group assignment at baseline to assist participants in their download of the correct app version. The assessor did provide each man with an orientation to the technology involved with his corresponding assigned app, either the premium or basic app version. Both intervention groups received a print version of their assigned app’s user manual that was adapted for this study. Data safety monitoring was conducted across all stages of the study.

### Interventions

#### Mobile technology plus arm (MT+)

Men randomly assigned to the MT+ arm received a premium version of a commercially available weight loss app (Lose-It!, FitNow Inc., Boston, MA) that allowed for real-time self-monitoring of eating and activity and included enhanced customization of personalized reports outlining self-monitoring trends and permitting personalized goal setting.

The premium app permitted men to participate in a private discussion board, consisting of men in the MT+ arm, allowing sharing of personal self-monitoring strategies and experiences. Men were encouraged to sign into to discussion board weekly where a trained, male moderator posted and facilitated weekly self-monitoring challenges. The men could respond to each other’s posts and post new comments or questions. Another feature of the premium app was its ability to sync weight as measured by a smart scale (Withings Body+ Composition Wi-Fi-enabled smart scale, Withings Inc., Cambridge, MA) to permit real time upload of daily weight and provide feedback regarding weight trends.

The MT+ group also received one-way text messages containing content on healthy eating and physical activity. Messages also provided prompts for self-monitoring. Texts were pushed one to two times daily (0800, 1100) and were delivered through the communication platform Remind.com (Remind Coaching, San Francisco, CA). The study’s community advisory board reviewed and provided feedback on the text message content to ensure local relevance.

Men in the MT+ group received 24-h internet technology troubleshooting support (ie. synching devices, lost Wi-Fi signal) through text or phone with a student nurse. The nurse also checked each participant’s app self-monitoring logs weekly for tracking frequency. Any man who did not log weight, eating, or physical activity for greater than 5 days received a positively worded reminder text. If there was no response to the text reminder, the nurse then provided a follow-up phone call reminder.

#### Mobile technology basic arm (MT)

Participants enrolled in the MT intervention received only the basic weight loss app from the same manufacturer (Lose-It! basic, FitNow Inc., Boston, MA). The basic version of this app is available at no cost and is widely accessible on smartphone app stores. The basic version app permitted daily real-time self-monitoring of weight via manual logging, eating and activity; yet this basic app did not provide insight trend reports of the user’s log content. Men in this arm did not receive any of the following: text message prompts for self-monitoring, Wi-Fi weight scale, within arm peer interaction for self-monitoring, investigator-initiated internet connection troubleshooting, or re-engagement support for intensively self-monitoring daily eating, activity, and weight logs. Participants in this arm received a text message reminder only as related to their upcoming assessment visit time and date.

### Assessments and outcome measures

A centrally located district public health department was the site for participant orientation and assessments. Participants from both groups were assessed at three time points: baseline, 3 months and 6 months. These time points corresponded to active weight loss (baseline to 3 months) and self-directed (3 months to 6 months).

A public health worker, trained by the investigators, conducted all aspects of the assessments including data collection and technology orientation for the men. The assessor received checklists and a manual with policies and procedures, and the investigators conducted fidelity checks for the assessor’s adherence to protocols. Sociodemographic characteristics of the participants and a brief health history were assessed via survey at baseline. The men’s experience with technology was evaluated at 3 and 6 months using the Comfort with Technology survey [24] at baseline, and the Technology Feasibility and Acceptability survey, adapted from the health-ITUES [38]. Blood pressure and pulse rate were collected at each time point using an automated machine following standardized methods [39].

### Feasibility and acceptability

Feasibility and acceptability were determined through participant retention counts, engagement/adherence with app logging and use, and focus group evaluation. Successful retention was defined as at least 70% of men who provided baseline measures, based upon previous men’s weight loss trials [16, 40].

Engagement and adherence with app logging was conducted for behaviors of weight tracking, and eating and activity behaviors. For weight, men were asked self-monitor their weight and log in their app every day. For the MT+ arm who received the Wi-Fi smart scale, recorded weights were synched to the premium app while men in the MT arm were instructed to use an accessible scale to weigh at home and manually enter their weight into their basic app log. Successful self-weighing adherence was defined as one or more recorded days per week [41, 42].

Dietary self-monitoring adherence was defined as recording a minimum amount (800 kcal) of caloric intake daily [41, 42]. Physical activity self-monitoring adherence was defined as recording total step count for 4 days or more per week [43]. Recorded counts of user app engagement were collected from participant’s tracking log.

Two focus groups were conducted with purposively selected MT+ completers of the 6 month intervention. They were stratified according to successful weight loss (≥5% from baseline) or unsuccessful weight loss (< 5% from baseline) at 6 months. Focus groups were led by a skilled moderator who solicited participant’s perceptions of the technology component’s (app, scale, text messaging, discussion board) acceptability and feasibility. Focus groups were audio recorded and transcribed verbatim [44]. Participants received a $25.00 stipend for their participation.

### Potential for weight loss and behavior change

Weight loss at 6 months was considered a preliminary primary outcome. Bodyweight in kilograms (kg) and height in centimeters were assessed using a digital electrical impedance scale (Tanita Model 215) that included a stadiometer following the manufacturer’s protocol [45]. Participants were asked to remove items (belts, tools, keys) from pockets and to remove shoes and socks prior to being weighed. Secondary outcomes reflecting diet and activity were conducted via survey instruments shown to have high reliability and validity including the Behavioral Risk Factor Surveillance Survey (BRFSS) Physical Activity Module [46], BRFSS Fruit and Vegetable Dietary Intake [47], and the Brief Questionnaire to Assess Beverage Intake (BEVQ-15) [48, 49].

### Analysis

The sample size was based upon currently accepted practice [31] for design and analysis of a pilot studies and estimation of effect size to inform power analysis of a future larger trial. Recommendations for 30 participants per group totaling 60 men, with allowances for up to a 25% attrition rate was used with a total planned enrollment of 80 men [50].

Feasibility of recruitment and retention was measured through counts of screened, enrolled, randomized, and retained for the duration of the intervention. Descriptive accounts of participant satisfaction were measured through usability surveys and focus group interviews. To measure engagement and logging adherence to the self-monitoring app, data from each participant’s account was downloaded as de-identified data and stored on an online research compliant platform.

Ninety-minute focus groups were held with MT+ participants to examine completer’s perceived satisfaction with the intervention components. Questions were asked such as: “Tell me about your experience with logging on the Lose-It! App?”, “How was your experience with receiving daily text messages?” and “How could the technologies in this study be adapted to make them more tailored to your work and lifestyle?” Qualitative descriptive content analysis [51] was used to reduce and interpret the focus group findings. Each focus group transcript constituted a unit of analysis permitting within case and then across case comparison. Topics of technology acceptability that were outlined in the interview guide were extracted a priori for substantial coding. Data corresponding to each question was coded together. Interviews were coded independently by two researchers and then compared and discussed as a team with an experienced qualitative researcher until consensus was reached. Coded data was entered into a data matrix to search for patterns across coding categories. Resulting categories were then compared against the entire study data set to determine enhanced explanation or interpretation of the outcome data.

Participant’s profiles were analyzed for descriptive statistics on outcomes by time point and arm. Those participants who met clinically significant threshold of weight loss (5% or more) were identified. Descriptive statistics were calculated on all demographic variables, and weight and healthy eating behavior outcomes by time point and by group. Change between baseline and 3 months, and baseline and 6 months outcomes were reported.

## Results

### Participant flow

**Fig. 1:**
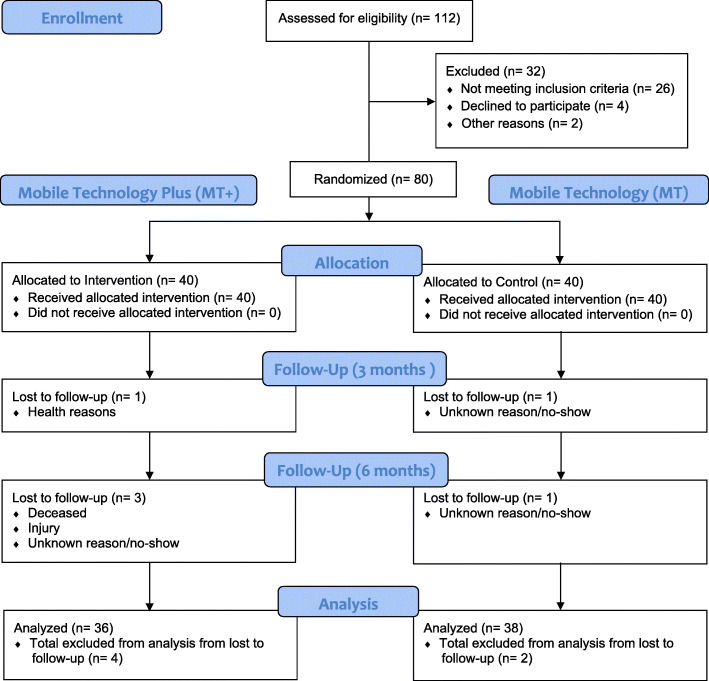
CONSORT 2010 participant flow diagram

A total of 80 participants (*n* = 40 MT+; *n* = 40 MT) were recruited, see Fig. 1 for the CONSORT flow diagram. A total of 112 men completed the eligibility screening process, 86 eligible men were screened, and 80 men were enrolled and randomized to groups. Of the 80 randomized participants, 39 (98%) MT+ and 39 (98%) usual care were assessed at 3 months. Of the total sample, 74 participants completed all three visit points, with an overall retention rate of 92.5%. The MT+ arm had 36 participants (90%) completion the study, and the MT arm 38 (95%). It should be noted however, that MT+ arm had two involuntary withdrawals, so reflected the same voluntary withdrawal rate as the MT group (Fig. 1).

### Baseline characteristics

Our participants were middle-aged (mean, [SD], 54.2 [8.6] years) and obese class 1 and 2, representing 27 and 33.8% in each class respectively (Table 1). The men were predominantly White/Non-Hispanic Latino descent 78 (97.5%), married 70 (88.6%) and obese 66 (82.6%). Overwhelmingly, the BMI scores fell into obesity class 1 (30 to < 35 kg/m^2^) and class 2 (35 to < 40 kg/m^2^) with similar numbers in the MT+ (*n* = 13, 16) and MT (*n* = 14, 11) respectively. While our cohort reflected diverse educational status, household incomes clustered at $60,000 or greater (Table 1). Most participants were employed, 72 (90%), and full time 65 (81.3%). Only 5 (6.3%) men were part-time, and one (1.3%) in the MT arm was retired. Although the majority 75 (96.2%) of participants were within 30 miles of a primary care provider, 18 (22.5%) had not seen their provider in the past year. Participants resided largely in rural (40%) and small town core (23.8%) locations [52].

**Table 1:** Baseline participant characteristics by arm and total (*n* = 80)

	MT+ ***n*** = 40	MT ***n*** = 40	Total ***n*** = 80
**Sociodemographic Characteristics**			
Age, years, mean ± SD	54.06 ± 8.15	54.35 ± 9.10	54.20 ± 8.59
**Clinical Characteristics**			
Weight, kg, mean ± SD	116.5 ± 19.56	111.63 ± 22.45	114.07 ± 21.07
BMI, kg/m^2^, mean ± SD	36.1 ± 6.43	35.79 ± 6.16	35.59 ± 6.91
Systolic BP, mmHg, mean ± SD	133.9 ± 11.69	134.7 ± 14.53	134.34 ± 13.11
Diastolic BP, mmHg, mean ± SD	81.69 ± 8.44	78.81 ± 10.48	80.25 ± 9.56
Pulse, beats per minute, mean ± SD	72.54 ± 12.09	68.3 ± 8.64	70.42 ± 10.66
**BMI Classifications, kg/m**^**2**^			
Overweight (25 to < 30)	6 (15.0%)	8 (20.0%)	14 (17.5%)
Obesity Class 1 (30 to < 35)	13 (32.5%)	14 (35.0%)	27 (33.8%)
Obesity Class 2 (35 to < 40)	16 (40.0%)	11 (27.5%)	27 (33.8%)
Obesity Class 3 (40 or higher)	5 (12.5%)	7 (17.5%)	12 (15.0%)
**Fluctuation of Weight in a Given Year**			
***n*** **= 78 (39, 39)**			
Weight, kg, mean ± SD	4.01 ± 2.59	4.07 ± 2.97	4.04 ± 2.77
**Dietary and Physical Activity Behaviors**			
Water intake, fluid ounce/day, mean ± SD	36.9 ± 17.05	36.7 ± 14.60	36.8 ± 15.78
SSB intake, fluid ounce/day, mean ± SD	9.28 ± 14.16	8.70 ± 13.10	8.99 ± 13.56
Fruits/Veg, serving/day, mean ± SD	2.15 ± 1.88	2.36 ± 1.35	2.26 ± 1.63
Activity, steps/day, mean ± SD, *n* = 76 (38, 38)	6235.85 ± 3772.65	6459.66 ± 3049.22	6347.75 ± 3408.98
**Distance from Fresh Produce (Fruits/Veg)**			
Less than 5 miles	27 (67.5%)	21 (52.5%)	48 (60%)
5–10 miles	9 (22.5%)	8 (20%)	17 (21.3%)
10–20 miles	2 (5%)	9 (2.5%)	11 (13.8%)
20–30 miles	1 (2.5%)	1 (2.5%)	2 (2.5%)
Greater than 30 miles	1 (2.5%)	1 (2.5%)	2 (2.5%)
**Race**			
White	40 (100%)	38 (95%)	78 (97.5%)
Black or African American	0 (0%)	1 (2.5%)	1 (1.3%)
Asian	0 (0%)	1 (2.5%)	1 (1.3%)
**Ethnicity**			
Not Hispanic/Latino	39 (97.5%)	39 (97.5%)	78 (97.5%)
Unknown	1 (2.5%)	1 (2.5%)	2 (2.5%)
**Marital Status,*n*** **= 79 (39, 40)**			
Single	1 (2.6%)	2 (5%)	3 (3.8%)
Married	34 (87.2%)	36 (90%)	70 (88.6%)
Widowed	1 (2.6%)	0 (0%)	1 (1.3%)
Divorced	3 (7.7%)	2 (5%)	5 (6.3%)
**Highest Level of Education**			
High school graduate/GED	3 (7.5%)	3 (7.5%)	6 (7.5%)
Some college but no degree	4 (10%)	9 (22.5%)	13 (16.3%)
Associates degree	6 (15%)	8 (20%)	14 (17.5%)
Bachelor’s degree	17 (42.5%)	7 (17.5%)	24 (30%)
Master’s degree	9 (22.5%)	9 (22.5%)	18 (22.5%)
Doctoral degree	1 (2.5%)	4 (10%)	5 (6.3%)
**2018 Household Income before Taxes**			
***n*** **= 78 (40, 38)**			
Under $20,000	1 (2.5%)	0 (0%)	1 (1.3%)
$20,000–$39,000	3 (7.5%)	3 (7.9%)	6 (7.7%)
$40,000–$59,000	3 (7.5%)	4 (10.5%)	7 (9%)
$60,000–$79,000	6 (15%)	10 (26.3%)	16 (20.5%)
$80,000–$99,000	11 (27.5%)	8 (21.1%)	19 (24.4%)
$100,000 or more	16 (40%)	13 (34.2%)	29 (37.2%)
**Employed**			
Yes	38 (95%)	34 (85%)	72 (90%)
No	2 (5%)	6 (15%)	8 (10%)
**Household Size including Participant**			
One	5 (12.5%)	1 (2.5%)	6 (7.5%)
Two	18 (45%)	17 (42.5%)	35 (43.8%)
Three or more	17 (42.5%)	22 (55%)	39 (48.9%)
**Health Insurance**			
***Government***			
Marketplace (ACA)	2 (5%)	1 (2.5%)	3 (3.8%)
Veterans Affairs or Military	2 (5%)	2 (5%)	4 (5%)
Medicare	2 (5%)	5 (12.5%)	7 (8.8%)
***Private***			
Health Maintenance Organization	0 (0%)	1 (2.5%)	1 (1.3%)
Private Insurance Company	34 (85%)	34 (85%)	68 (85%)
***Unsure/Unknown***			
Don’t Know/Not Sure	1 (2.5%)	0 (0%)	1 (1.3%)
None	0 (0%)	1 (2.5%)	1 (1.3%)
**Distance from Primary Care Provider**			
***n*** **= 78 (38, 40)**			
Less than 5 miles	21 (55.3%)	19 (47.5%)	40 (51.3%)
5–10 miles	9 (23.7%)	4 (10%)	13 (16.7%)
10–20 miles	4 (10.5%)	6 (15%)	10 (12.8%)
20–30 miles	3 (7.9%)	9 (22.5%)	12 (15.4%)
Greater than 30 miles	1 (2.6%)	2 (5%)	3 (3.8%)
**Last Time Seen by a Healthcare Provider**			
Less than a week	1 (2.5%)	4 (10%)	5 (6.3%)
2–3 weeks	4 (10%)	3 (7.5%)	7 (8.8%)
1–3 months	6 (15%)	9 (22.5%)	15 (18.8%)
3–6 months	11 (27.5%)	9 (22.5%)	20 (25%)
6–12 months	8 (20%)	6 (15%)	14 (17.5%)
Over a year	9 (22.5%)	9 (22.5%)	18 (22.5%)
Unknown	1 (2.5%)	0 (0%)	1 (1.3%)

Note:

a. Abbreviations: *ACA* Affordable Care Act; *BMI* body mass index; *BP* blood pressure; *kg* kilogram; *MT+* mobile technology plus intervention; *MT* basic mobile technology; *SD* standard deviation; *SSB* sweetened sugar beverages; *Veg* vegetables

b. * *p* < 0.05 ** *p* < 0.01 *** *p* < 0.001

c. *n* = 80 unless specified as *n* = # of total (# of MT+, # of MT)

### Feasibility and acceptability

Feasibility and acceptability of the intervention with rural men was explored by comparing the averages, percentages, and proportions of the adherence to tracking weight, dietary intake, and physical activity. A general trend of higher adherence rates for each category was observed in both arms in the first 3 months (active weight loss phase) of the study compared to the second 3 months (self-directed phase); yet key differences were found (Table 2). Exploration into whether age of the participant contributed to a difference was examined by separating the men into two age groups (40–59; 60 and older).

**Table 2:** Feasibility and acceptability of MT+ and MT in a rural men’s weight loss study

Measures	Criterion Met?	Assessment
Demonstrate feasibility of recruiting 80 men in a rural setting and retaining at least 70% of men who provided baseline measures	Yes	Over 10 months, 80 rural men living in RUCA codes 4–10 were recruited and matriculated to baseline assessment after informed consent. At baseline, 100% of the 80 men provided baseline measurements. At 3 months, 78 (97.5%) provided measurements. At 6 months, 74 (92.5%) provided measurements.
Evidence to suggest that recording weight one or more days per week on the Lose it! app was feasible and acceptable by participants.	Yes, during the active weight loss phase	The active weight loss phase (AWLP; baseline to 3-months) showed the highest retention rates for measuring weights at least once a week. For both MT+ and MT, participants recorded self-weights at least once a week at a rate of 64.1% (AWLP) but this decreased to 46.3% during the self-directed phase (SDP; between 3- and 6 months). For the MT+ arm, 84.6% weighed themselves at least once a week or more during AWLP, but this decreased to 59.4% during SDP. For the MT arm, 43.5% weighed themselves at least once a week or more during AWLP, but this decreased to 33.5% during SDP.
Evidence to suggest that recording a minimum amount of 800 kcal 5 days or more per week to monitor dietary intake was feasible and acceptable by participants.	Yes, during the active weight loss phase	The highest retention rates with recording 800 dietary calories at least 5 days or more every week were noted during the AWLP than the SDP. For both MT+ and MT, participants recorded 800 dietary calories at least 5 days a week at a rate of 73.4% (AWLP) but this decreased to 51.6% during SDP. For the MT+ arm, 76.0% recorded 800 dietary calories at least 5 days or more every week AWLP, but this decreased to 55.2% during SDP. For the MT arm, 70.8% recorded 800 dietary calories at least 5 days or more every week during AWLP, but this decreased to 47.9% during SDP.
Evidence to suggest that recording a total step count for 4 days or more per week for physical activity levels was feasible and acceptable by participants.	Yes	For total step counts, both the MT+ and MT groups averaged recording at least 6 days each week at each time point. The MT+ participants recorded total steps on average (SD), 6.74 (0.86), 6.86 (0.41) and 7 (0.0) days per week at baseline, 3 months, and 6 months, respectively. For the MT arm, participants recorded total steps on average, 6.84 (0.44), 6.52 (1.28), and 6.76 (0.83) days per week at baseline, 3 months, and 6 months, respectively.
Evaluation to suggest acceptability of the Lose it! app by participant’s self-report of acceptability of the app, scale, text messaging and discussion board.	Partially	MT+ Lose it! app was supportive in assisting participants to lose weight and is an accessible means to monitor eating, activity, and weight in rural locations. Participants voiced a desire to having an increase in the intensity of support (another weight goal) after achieving their personal weight loss goal.

Note:

a. Abbreviations: *app* application; *MT+* mobile technology plus; *MT* basic mobile technology, *AWLP* active weight loss phase; *RUCA* rural-urban commuting area; *SD* standard deviation; *SDP* self-directed phase

Self-monitoring weight

Participant adherence to self-monitoring was determined via logging of weight once a week or more. Generally, participants in both arms demonstrated a higher percentage of weighing at least once a week in the active weight loss phase compared to the self-directed phase (Table 3).

**Table 3:** Self-monitoring of weight, dietary intake, and activity by arm

Variable	Mobile Technology Plus (MT+)		Mobile Technology Basic (MT)		Total	
	n (%)^**a**^	Mean (weeks)	n (%)	Mean (weeks)	n (%)	Mean (weeks)
**Weight**^**b**^						
Baseline to 3 months	40 (84.6%)	10.2	40 (43.5%)	5.2	80 (64.1%)	7.7
3 months to 6 months	39 (59.4%)	7.1	40 (33.5%)	4.0	79 (46.3%)	5.6
**Dietary Intake**^**c**^						
Baseline to 3 months	40 (76.0%)	9.1	40 (70.8%)	8.5	80 (73.4%)	8.8
3 months to 6 months	40 (55.2%)	6.6	40 (47.9%)	5.8	80 (51.6%)	6.2
**Weekly Activity Reporting**^**d**^	**Mean (SD)**^**a**^		**Mean (SD)**		**Mean (SD)**	
Baseline	6.74 (0.86)		6.84 (0.44)		6.79 (0.68)	
3 months	6.87 (0.41)		6.52 (1.28)		6.71 (0.94)	
6 months	7 (0.0)		6.76 (0.83)		6.89 (0.58)	

Note:

a. Abbreviations: *SD* standard deviation, *n* number of subjects who completed, *%* percent of total sample

b. ^b^Weight- self measurement logged at least once weekly

c. ^c^Dietary Intake- self measurement of at least 800 cal daily logged a minimum of 5 days per week

d. ^d^Days per week activity steps were measured by Lose it! Smartphone application

In the MT+ arm, 84.6 and 59.4% of men recorded a weight at least once a week during the active weight loss phase and self-directed phase, respectively. This averages to recording a weight at least once a week for 10.2 and 7.1 weeks, during months 1–3 and 3–6, respectively. Conversely, in the MT arm, 43.5 and 33.5% of men recorded a weight at least once a week during the active weight loss phase and self-directed phase, respectively.

#### Dietary intake self-monitoring

Participants recording 800 cal each day for at least 5 days or more in a week were considered adherent to dietary self-monitoring. Overall, participants in both arms demonstrated a higher percentage of tracking 800 cal each day at minimum 5 days a week in the app during the active weight loss phase (baseline to 3 months) compared to the self-directed phase (3 to 6 months) (Table 3). In the MT+ arm, 76.0 and 55.2% recorded 800 cal for at least 5 days each week in the active and self-directed weight loss phases, respectively. This averages to recording 800 cal at least 5 days each week for 9.1 and 6.6 weeks, for months 1–3 and 3–6, respectively. On the other hand, the MT arm recorded dietary intake at a rate of 70.8% during the 1st 3 months and 47.9% between months 3 and 6. The high adherence rate to participant tracking 70% MT+ supports the app is feasible and acceptable. Statistics of re-engagement attempts for self-monitoring adherence in the MT+ arm also supported feasibility and acceptability. Eleven men required re-engagement for not logging their eating, activity or weight. Of these men, 36% (*n* = 4) required one contact, 55% (*n* = 6) received two contacts, and 9% (*n* = 1) required three contacts before returning to consistent logging of their daily self-monitoring.

#### Self-monitoring physical activity

Data obtained from both groups indicated that men regularly wore their smartphones. For the MT+ arm, participant’s smartphone devices recorded steps of an average of 6.74 (0.86), 6.86 (0.41) and 7 (0.0) days per week across all time points (baseline, 3 months, and 6 months), respectively. For the MT arm, averages were 6.84 (0.44), 6.52 (1.28), and 6.76 (0.83) days per week at baseline, 3 month and 6 months, respectively.

#### Acceptability

Acceptability of the Lose-It! app, smart scale, discussion group and text messages were evaluated by administering descriptive surveys and through focus groups. Overall, participants in both groups reported the use of smartphone and internet-based technologies as accessible and familiar. At baseline, the men were asked survey questions regarding their comfort level with technology use. Even while living in rural locations, all men stated having access to high-speed or broadband internet at home and only 66 (90.4%) were aware of having internet at work. Men commented using the internet on their smartphones several times daily (56.3%) and daily (38.8%). The top three reasons cited for using smartphone apps were weather (77.5%), news (62.5%) and sports/entertainment (58.8%). Many participants used their smart phones to text (100%), access the internet (98.8%), to check email (95%), and to use global positioning system (GPS, 92.5%); most highly rated their comfort levels with these stated apps as very comfortable and comfortable.

#### Health-ITUES

Participants in the MT+ and MT overall strongly agreed to agreed that the app would be a positive addition in assisting rural men to lose weight (79.5, 72.3%). The majority of MT+ and MT participants were agreeable that the app improved quality of life (76.9, 92.3%) and is an important part in meeting informational needs (76.9, 87.2%). In the MT+ arm, satisfaction rates with the app and smart scale app were high: self-monitoring weight (94.7, 97.2%), eating (92.1, 91.7%) and physical activity (76.3, 83.3%) at 3 and 6 months respectively. In the MT arm, participants reported satisfaction of 97.4 and 91.9% (3 and 6 months) with the basic app in helping to self-monitor weight, eating, and physical activity. A lower but favorable appraisal of text messages was elicited from the MT+ participants. They agreed that text messages increased their confidence to self-monitor weight (68.4, 58.3%), eating (68.4, 69.4%) and physical activity (50, 50%) at 3 and 6 months, respectively. The MT+ participants neither agreed nor disagreed when asked if the online discussion group increased confidence in self-monitoring weight, eating and physical activity at both 3- and 6 months. While a descriptive narrative summary of the discussion board acceptability was clarified by MT+ participants during the focus groups, counts of participant posts and their descriptive categories were also noted. A total of 14 MT+ participants posted at least once on the discussion board for a total of 45 posts. The 45 posts ranged in content from topics of: strategy sharing (58%), motivational (16%), humor (13%), self-monitoring frustrations (9%), and behavior evaluation (4%).

Two focus groups comprised of MT+ completers were held. One group comprised of successful MT+ completers (*n* = 6, experience of 5% or greater baseline body weight loss) and unsuccessful MT+ completers (*n* = 5, experience of less than 5% loss of baseline body weight). The central topic discerned consistently across groups was intervention intensity. Both groups found the MT+ components supportive to achieving weight loss and an accessible means to self-monitor eating, activity, and weight in rural settings. Successful participants reported high level engagement with self-monitoring weight, eating and activity. However, they wanted heightened intervention intensity (boosters) after their personal weight loss goal was met to help them maintain motivation for ongoing self-monitoring. Both successful and unsuccessful groups reported a general dislike for the asynchronous nature and low posting frequency of the discussion board. Specifically, participants found the post replies from other participants too infrequent, which led to feelings of isolation and frustration. Participants described the sporadic peer posting activity on the asynchronous discussion board as frustrating which decreased their motivation to sign into it regularly. Many participants described reading other participant’s discussion postings but not posting themselves. Participants felt that synchronous discussion boards would encourage all participants to engage in back and forth dialogue better than an asynchronous format. Both groups also felt that real-time discussion would increase feelings of support. Unsuccessful participants reported lower engagement in self-monitoring intensity (logging every few days rather than after each meal) and greater difficulty in navigating the technologies. Unsuccessful weight loss participants clarified potential reasons for nonadherence to self-monitoring, such as competing demands of job prohibiting engaging in real time self-monitoring until end of day, or a lifestyle pattern of retiring their phone (not keeping it on them) at the end of the workday, which decreased their likelihood to log evening intake or activity.

Some unsuccessful participants voiced a positive outcome of not experiencing their typical seasonal weight gain of 9–14 kg during the intervention, and stated they considered their body weight maintenance as a success outcome of study participation. Both groups desired live and personalized social support in the form of professional and peer coaching as an important component to engagement. The asynchronous discussion board format was not desired by either group who found it impersonal, instead desiring real-time support and coaching face to face or via phone.

#### Weight and behavioral measures

Observed means and behavioral measures are reported in Table 4.

**Table 4:** Observed means of outcome measures by arm

Variable	Observed Means			
	Mobile Technology Plus (MT+)		Mobile Technology Basic (MT)	
	***n*** = 40		***n*** = 40	
	n	Mean (SD)	n	Mean (SD)
**Fruits/Vegetables (serving/day)**				
Baseline	40	2.15 (1.88)	40	2.36 (1.35)
3 months	39	2.90 (1.67)	38	2.71 (1.17)
6 months	36	3.16 (1.67)	38	3.09 (1.54)
**Water intake**^**a**^**(fluid oz/day)**				
Baseline	40	36.9 (17.05)	40	36.7 (14.60)
3 months	39	38.05 (13.85)	38	34.95 (17.11)
6 months	36	37.11 (16.47)	37	34.7 (15.15)
**Sweetened Beverage intake**^**a**^**(fluid oz/day)**				
Baseline	40	9.28 (14.16)	40	8.70 (13.10)
3 months	39	7.43 (11.33)	38	6.22 (7.48)
6 months	36	4.34 (6.44)	38	10.58 (17.01)
**Activity**^**b**^**(steps/day)**				
Baseline	38	6235.85 (3772.65)	38	6459.66 (3049.22)
3 months	37	6576.90 (4189.11)	34	6252.55 (3776.22)
6 months	28	6874.64 (4650.58)	25	5359.50 (2995.44)
**Systolic Blood Pressure (mmHg)**				
Baseline	40	133.9 (11.69)	40	134.7 (14.53)
3 months	39	126.69 (12.04)	39	130.76 (14.4)
6 months	35	124.6 (11.60)	38	127.57 (11.91)
**Diastolic Blood Pressure (mmHg)**				
Baseline	40	81.69 (8.44)	40	78.81 (10.48)
3 months	39	76.79 (8.6)	39	76.99 (8.39)
6 months	35	75.47 (10.62)	38	76.41 (9.32)
**Pulse (beats per minute)**				
Baseline	40	72.54 (12.09)	40	68.3 (8.64)
3 months	39	68.12 (12.85)	39	66.97 (9.14)
6 months	35	66.81 (9.83)	38	65.59 (8.66)

Note:

a. Abbreviations: *BMI* body mass index; *oz* ounce

b. ^a^*BEVQ-15* Brief Questionnaire to Assess Beverage Intake

c. ^b^Activity steps as measured by Lose it! Smartphone application

**Fig. 2:**
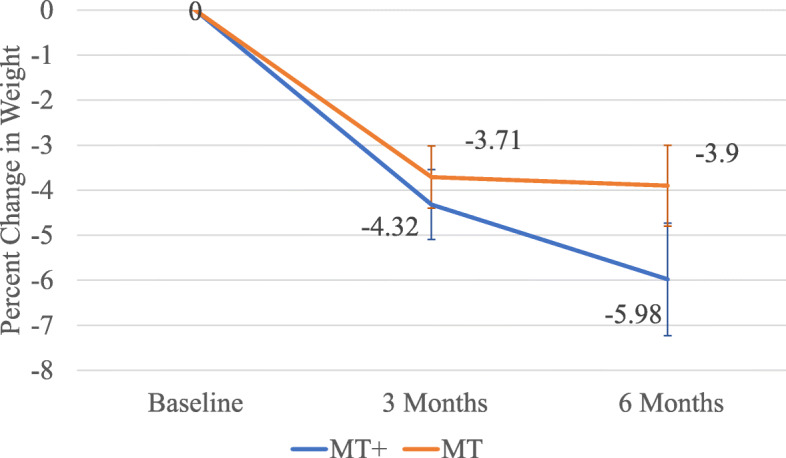
Percent mean weight change ± SE by time and intervention arm over 6 months. (Legend: MT+, mobile technology plus intervention, MT, basic mobile technology)

Mean weight loss across groups ranged from 4.8 kg gained to 43.5 kg lost, representing from 0 to 29% of initial weight (Fig. 2).

**Fig. 3:**
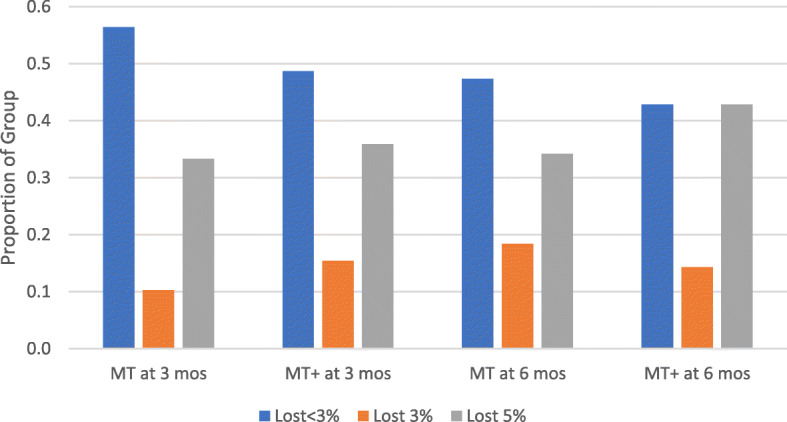
Percent weight loss proportional by intervention arm at 3 months and 6 months. (Legend: MT+, mobile technology plus intervention, MT, basic mobile technology, mos, months)

Overall, 40/73 (54.8%) men lost ≥3%; 28/73 men (38.4%) lost ≥5% body weight; 12/73 (16.4%) lost between 1 and 3%; and 21/73 (28.8%) were below 1% lost, with 15 of those being weight gainers (Fig. 3).

Total percent body weight loss for the MT arm averaged 3.9% and the MT+ arm was 5.98% respectively (see Table 5).

**Table 5:** Mean change in BMI, weight, and percentage from baseline measurements by randomized group

Outcome Variable	MT+ Mean ± SD	MT Mean ± SD	Difference between groups Mean ± SE
**Change in BMI**			
At 3 months	−1.4 ± 1.75	−1.19 ± 1.61	0.21 ± 0.38
Between 3 and 6 months	−0.61 ± 1.62	−0.14 ± 1.16	0.47 ± 0.33
At 6 months	−2.17 ± 2.9	− 1.36 ± 1.9	0.81 ± 0.57
**Weight change, kg**			
At 3 months	−5.06 ± 5.83	−3.88 ± 4.52	1.17 ± 1.18
Between 3 and 6 months	−1.5 ± 4.98	−0.16 ± 3.91	1.34 ± 1.04
At 6 months	−7.03 ± 9.78	−4.14 ± 5.85	2.89 ± 1.87
**Weight change, %**			
At 3 months	−4.32 ± 4.65	−3.71 ± 4.27	0.61 ± 1.01
Between 3 and 6 months	− 1.44 ± 4.35	−0.12 ± 3.57	1.32 ± 0.93
At 6 months	− 5.98 ± 7.5	−3.9 ± 5.53	2.08 ± 1.53

Note:

a. Abbreviations: *BMI* body mass index; *kg* kilogram; *MT+* mobile technology plus intervention; *MT* basic mobile technology; *SD* standard deviation; *SE* standard error

Analysis of BRFSS survey data for self-reporting activity was not possible due to a branching logic error in REDCap. Although no significant effects were observed for the primary outcome of weight or average steps per day, trends were in the expected directions. Although no significant effects were observed in the amount (fluid oz.) and energy (kcals) of daily water intake, Sugar Sweetened Beverage (SSB) and alcohol intake, favorable outcomes were observed. In the MT+ group, the intake of water increased and stayed above baseline values. A decrease in reported SSB intake and thus kilocalories was observed at the 3 month follow-up for both groups. However, the MT+ continued to reduce SSB intake while the MT showed an increase higher than baseline values at post-intervention. Alcohol intake decreased for both groups at 3 months and 6 months.

## Discussion

Our findings demonstrate that recruiting and retaining rural men into a community-based weight loss trial using mobile technologies is feasible. The levels of engagement and adherence to self-monitoring weight and dietary intake in the mobile app indicate that rural men in our study found the Lose-It! app acceptable. The achievement of clinically significant [53] weight loss by both groups at 6 months affirms that commercially available weight loss apps, such as Lose-It! are feasible in engaging rural men by providing goal setting and access to real-time feedback. Our finding that the MT+ participants achieved higher engagement, as measured by Lose-It! log entries between baseline and 3 months, suggest that the text messaging, logging engagement prompts, and supplementary feedback support improved self-monitoring adherence. Acceptability survey and focus group feedback also confirmed our observations. Our findings are consistent with previous studies, including those tailored to minority populations [26], who found tailored interventions utilizing self-monitoring app components led to higher rates of self-monitoring engagement [54, 55].

Weekly tracking of weight is an integral part of maintaining engagement with one’s self-monitoring plan for successful weight loss [56]. The smart scale appeared to provide dual researcher-participant benefit, with an objective measure of self-weighing frequency and additional prompting source supporting self-monitoring adherence. While app self-monitoring prompts were embraced by participants, the app-based discussion group had limited acceptability. Social support was identified as an important component of ongoing engagement in self-monitoring [57]; however, from our rural participant’s perspective, it must be in real time, personalized, and promote interaction that creates a community reciprocal support. This points to the potential for multicomponent peer support via live video or phone conferencing in addition to online support as worthwhile components for future testing. This is consistent with the current weight loss literature reporting peer group preferences in men [58]. Looking forward, we will adapt this intervention to include more contextually sensitive social support components in a fully powered study.

Both groups showed improvements in weight reduction reporting lower values for weight and BMI at 3 and 6 months post baseline. Both groups self-monitored eating and activity using the same core self-monitoring functions of the Lose-It app (logging caloric intake and physical activity). Self-monitoring apps, such as Lose-It! have established effectiveness for weight loss when used consistently [59, 60]. Both groups in our study achieved averages of ≥3% bodyweight loss from baseline, which is considered as clinically significant weight loss [53, 61]. A respectable proportion of completers achieved averages of ≥5% weight loss, considered clinically relevant for health across both arms: MT+ arm, 42.9% (15/35) and MT arm, 34.2% (13/38) [53] [61]. The MT+ group averaged higher percent weight loss at 6 months (see Table 5), suggesting that the enhanced personalization of the MT+ features derived from community-engaged strategies (individualized feedback in several forms and multiple forms of peer support) may be important engagement factors for future interventions [58, 60]. Our findings follow a similar pattern described in the weight loss literature. Specifically that technology-based weight loss interventions provide multiple feedback forms supporting greater self-monitoring adherence [27]. Our findings may also reflect age cohort differences in mobile health technology engagement that can serve as a next step towards a future intervention to modify remote, mobile health self-monitoring behavior of eating, activity, and weight for weight loss.

Limitations exist that necessitate caution in interpreting the results. This sample consisted of rural men who owned a smartphone and therefore may not reflect men with older mobile phone technology (flip phones). Our sample also consisted of moderate to high income and education level of participants which may limit generalizability. Future work should include the use of participatory strategies to engage rural men of lower education, income, and socioeconomic status, as well as examine the use of a demographic measure that more comprehensively encompasses the complex variables of poverty. Our weight loss intervention was 12 weeks long, which is short of duration to assess longer term feasibility and/or acceptability of the interventions. A standardized self-monitoring measure for adherence is not known and therefore different rates may be observed using different criteria [62]. Future work applying implementation science frameworks (e.g. RE-AIM, iPharis) will permit planning and integration of those intervention elements critically important for self-monitoring engagement with rural men. These frameworks can also help discern the decision making processes rural men use when determining program applicability to their rural context [63].

## Conclusion

In conclusion, this study illustrates that rural men can be recruited and retained into community-based clinical weight loss trials. Our findings suggest that self-monitoring with commercial-based apps can lead to clinically significant weight loss for rural men. Further study is needed to determine what combination of intervention components promote intensive self-monitoring engagement over time. While the presence of a self-monitoring app increases accessibility to rural men, additional intervention components such as text messaging and personalized coaching are important for sustained engagement. Mobile technology interventions show promise for understanding rural men’s engagement and adherence patterns for self-monitoring weight, eating, and activity for achieving weight loss.

## Data Availability

The dataset analyzed for this report is available from the corresponding author on reasonable request.

## Abbreviations

APP: Application
BEVQ-15: Beverage intake questionnaire 15 item
BMI: Body mass index
BRFSS: Behavioral risk factor surveillance survey
CAB: Community advisory board
GPS: Global positioning system
Kg: Kilograms
MT: Mobile technology
MT +: Mobile technologies plus
PAR-Q17: Physical activity readiness questionnaire 17 item
USA: United States of America
e.g.: For example
DSMP: Data safety monitoring board
REDCap: Research electronic data capture
HIPAA: Health insurance portability and accountability act
UNMC: University of Nebraska medical center

## References

[1] Hales C, Carroll M, Fryar C, Ogden C (2017). Prevalence of obesity among adults and youth: United States, 2015–2016.

[2] Ogden CL, Carroll MD, Kit BK, Flegal KM (2014). Prevalence of childhood and adult obesity in the United States, 2011-2012. JAMA..

[3] Bixby H, Bentham J, Zhou B, Di Cesare M, Paciorek CJ, Bennett JE (2019). Rising rural body-mass index is the main driver of the global obesity epidemic in adults. Nature..

[4] Guo Z, Jiang Y, Huffman SK. Agricultural mechanization and BMI for rural workers: a field experiment in China. Economics Working Papers: Department of Economics, Iowa State University. 2018;(18010):1-23.

[5] Pickett W, King N, Lawson J, Dosman JA, Trask C, Brison RJ, Hagel L, Janssen I, Saskatchewan Farm Injury Cohort Study Team (2015). Farmers, mechanized work, and links to obesity. Prev Med.

[6] NCD Risk Factor Collaboration (NCD-RisC). Rising rural body-mass index is the main driver of the global obesity epidemic in adults. Nature. 2019;569(7755):260-4. 10.1038/s41586-019-1171-x. 10.1038/s41586-019-1171-x PMC6784868 31068725

[7] Rural Health Report Policy Research Center. Exploring rural and urban mortality differences, August 2015. Bethesda; 2015.

[8] Shelton JB, Rajfer J (2012). Androgen deficiency in aging and metabolically challenged men. Urol Clin North Am.

[9] Goldstein SP, Goldstein CM, Bond DS, Raynor HA, Wing RR, Thomas JG (2019). Associations between self-monitoring and weight change in behavioral weight loss interventions. Health Psychol.

[10] Anderson-Lewis C, Darville G, Mercado RE, Howell S, Di Maggio S (2018). mHealth technology use and implications in historically underserved and minority populations in the United States: systematic literature review. JMIR Mhealth Uhealth..

[11] Podina IR, Fodor LA (2018). Critical review and meta-analysis of multicomponent behavioral e-health interventions for weight loss. Health Psychol.

[12] Beleigoli AM, Andrade AQ, Cançado AG, Paulo MN, Diniz MFH, Ribeiro AL. Web-based digital health interventions for weight loss and lifestyle habit changes in overweight and obese adults: systematic review andmeta-analysis. J Med Internet Res. 2019;21(1):e298. 10.2196/jmir.9609. 10.2196/jmir.9609 PMC6330028 30622090

[13] Han M, Lee E (2018). Effectiveness of mobile health application use to improve health behavior changes: a systematic review of randomized controlled trials. Healthc Inform Res.

[14] Ferrara G, Kim J, Lin S, Hua J, Seto E (2019). A focused review of smartphone diet-tracking apps: usability, functionality, coherence with behavior change theory, and comparative validity of nutrient intake and energy estimates. JMIR mHealth uHealth..

[15] Pagoto S, Schneider K, Jojic M, DeBiasse M, Mann D (2013). Evidence-based strategies in weight-loss mobile apps. Am J Prev Med.

[16] Robertson C, Avenell A, Stewart F, Archibald D, Douglas F, Hoddinott P, van Teijlingen E, Boyers D (2017). Clinical effectiveness of weight loss and weight maintenance interventions for men: a systematic review of men-only randomized controlled trials (the ROMEO project). Am J Mens Health.

[17] Pew Research Center. Demographics of mobile device ownership and adoption in the United States. 2021 [Available from: https://www.pewresearch.org/internet/fact-sheet/mobile/.

[18] Hu R, van Velthoven MH, Meinert E (2020). Perspectives of people who are overweight and obese on using wearable Technology for Weight Management: systematic review. JMIR mHealth uHealth.

[19] Lemon SC, Rosal MC, Zapka J, Borg A, Andersen V (2009). Contributions of weight perceptions to weight loss attempts: differences by body mass index and gender. Body Image.

[20] French SA, Jeffery RW, Wing RR (1994). Sex differences among participants in a weight-control program. Addict Behav.

[21] Lovejoy JC, Sainsbury A (2009). Stock conference working group. Sex differences in obesity and the regulation of energy homeostasis. Obes Rev.

[22] Nothwehr F, Snetselaar L, Wu H (2006). Weight management strategies reported by rural men and women in Iowa. J Nutr Educ Behav.

[23] Hiebert B, Leipert B, Regan S, Burkell J. Rural men's health, health information seeking, and gender identities: a conceptual theoretical review of the literature. Am J Mens Health. 2018;12(4):863-76. 10.1177/1557988316649177. 10.1177/1557988316649177 PMC6131457 27170674

[24] Eisenhauer CM, Hageman PA, Rowland S, Becker BJ, Barnason SA, Pullen CH (2017). Acceptability of mhealth technology for self-monitoring eating and activity among rural men. Public Health Nurs.

[25] Klitzman P, Armstrong B, Janicke DM. Distance as a predictor of treatment attendance in a family based pediatric weight management program in rual aeas. J Rural Health. 2015; 31(1):[19–26 pp.]. Available from: https://onlinelibrary.wiley.com/doi/full/10.1111/jrh.12078. 10.1111/jrh.12078 25040534

[26] Rosas LG, Lv N, Xiao L, Lewis MA, Venditti EMJ, Zavella P, Azar K, Ma J (2020). Effect of a culturally adapted behavioral intervention for Latino adults on weight loss over 2 years. JAMA Netw Open.

[27] Rumbo-Rodríguez L, Sánchez-Sansegundo M, Ruiz-Robledillo N, Albaladejo-Blázquez N, Ferrer-Cascales R, Zaragoza-Martí A (2020). Use of technology-based interventions in the treatment of patients with overweight and obesity: a systematic review. Nutrients..

[28] Serrano KJ, Coa KI, Yu M, Wolff-Hughes DL, Atienza AA (2017). Characterizing user engagement with health app data: a data mining approach. Transl Behav Med.

[29] Appel LJ, Clark JM, Yeh H-C, Wang N-Y, Coughlin JW, Daumit G, Miller ER, Dalcin A, Jerome GJ, Geller S, Noronha G, Pozefsky T, Charleston J, Reynolds JB, Durkin N, Rubin RR, Louis TA, Brancati FL (2011). Comparative effectiveness of weight-loss interventions in clinical practice. N Engl J Med.

[30] Yang Q, Van Stee SK (2019). The comparative effectiveness of mobile phone interventions in improving health outcomes: Meta-analytic review. JMIR mHealth uHealth..

[31] Whitehead AL, Sully BG, Campbell MJ (2014). Pilot and feasibility studies: is there a difference from each other and from a randomised controlled trial?. Contemp Clin Trials.

[32] Lancaster GA, Dodd S, Williamson PR (2004). Design and analysis of pilot studies: recommendations for good practice. J Eval Clin Pract.

[33] Eisenhauer CM, Brito FA, Yoder AM, Kupzyk KA, Pullen CH, Salinas KE, Miller J, Hageman PA (2020). Mobile technology intervention for weight loss in rural men: protocol for a pilot pragmatic randomised controlled trial. BMJ Open.

[34] Health Resources and Service Administration FOoR, Health Policy aUSDoA, Economic Research Service. Rural-urban commuting area codes (RUCAs). 2016.

[35] Goodman JM, Thomas SG, Burr J (2011). Evidence-based risk assessment and recommendations for exercise testing and physical activity clearance in apparently healthy individuals. Appl Physiol Nutr Metab.

[36] Thompson PD, Arena R, Riebe D, Pescatello LS (2013). ACSM’s new preparticipation health screening recommendations from ACSM’s guidelines for exercise testing and prescription. Curr Sports Med Rep.

[37] Hageman PA, Pullen CH, Hertzog M, Pozehl B, Eisenhauer C, Boeckner LS (2017). Web-based interventions alone or supplemented with peer-led support or professional email counseling for weight loss and weight maintenance in women from rural communities: results of a clinical trial. J Obes.

[38] Schnall R, Cho H, Liu J (2018). Health information technology usability evaluation scale (health-ITUES) for usability assessment of mobile health technology: validation study. JMIR Mhealth Uhealth.

[39] Pickering TG, Hall JE, Appel LJ, Falkner BE, Graves J, Hill MN, Jones DW, Kurtz T, Sheps SG, Roccella EJ, Subcommittee of Professional and Public Education of the American Heart Association Council on High Blood Pressure Research (2005). Recommendations for blood pressure measurement in humans and experimental animals. Hypertension..

[40] Morgan PJ, Callister R, Collins CE, Plotnikoff RC, Young MD, Berry N, McElduff P, Burrows T, Aguiar E, Saunders KL (2013). The SHED-IT community trial: a randomized controlled trial of internet- and paper-based weight loss programs tailored for overweight and obese men. Ann Behav Med.

[41] Tsai CC, Lee G, Raab F, Norman GJ, Sohn T, Griswold WG, Patrick K (2007). Usability and feasibility of PmEB: a mobile phone application for monitoring real time caloric balance. Mobile Netw Appl.

[42] Steinberg DM, Tate DF, Bennett GG, Ennett S, Samuel-Hodge C, Ward DS (2013). The efficacy of a daily self-weighing weight loss intervention using smart scales and e-mail. Obesity (Silver Spring).

[43] Troiano RP, Berrigan D, Dodd KW, Mâsse LC, Tilert T, McDowell M (2008). Physical activity in the United States measured by accelerometer. Med Sci Sports Exerc.

[44] Morgan D (2010). Reconsidering the role of interaction in analyzing and reporting focus groups. Qual Health Res.

[45] Tsui EYL, Gao XJ, Zinman B (1998). Bioelectrical impedance analysis (BIA) using bipolar foot electrodes in the assessment of body composition in type 2 diabetes mellitus. Diabet Med.

[46] Brownson RC, Jones DA, Pratt M, Blanton C, Heath GW (2000). Measuring physical activity with the behavioral risk factor surveillance system. Med Sci Sports Exerc.

[47] Centers for Disease Control and Prevention. Surveillance of fruit and vegetable intake using the behavioral risk factor surveillance system. 2015. Available from: https://www.cdc.gov/brfss/pdf/fruits_vegetables.pdf.

[48] Hedrick VE, Savla J, Comber DL, Flack KD, Estabrooks PA, Nsiah-Kumi PA, Ortmeier S, Davy BM (2012). Development of a brief questionnaire to assess habitual beverage intake (BEVQ-15): sugar-sweetened beverages and total beverage energy intake. J Acad Nutr Diet.

[49] Hedrick VE, Comber DL, Estabrooks PA, Savla J, Davy BM (2010). The beverage intake questionnaire: determining initial validity and reliability. J Am Diet Assoc.

[50] Hertzog MA (2008). Considerations in determining sample size for pilot studies. Res Nurs Health.

[51] Hsieh H-F, Shannon SE (2005). Three approaches to qualitative content analysis. Qual Health Res.

[52] United States Department of Agriculture Economic Research Service. Rural-urban commuting area codes: United States Department of Agriculture; 2020 [Available from: https://www.ers.usda.gov/data-products/rural-urban-commuting-area-codes.aspx.

[53] American Diabetes Association (2019). Obesity management for the treatment of type 2 diabetes: standards of medical care in diabetes 2019. Diabetes Care.

[54] Frie K, Hartmann-Boyce J, Jebb S, Oke J, Aveyard P (2020). Patterns in weight and physical activity tracking data preceding a stop in weight monitoring: observational analysis. J Med Internet Res.

[55] Alencar M, Johnson K, Gray V, Mullur R, Gutierrez E, Dionico P (2020). Telehealth-based health coaching increases m-health device adherence and rate of weight loss in obese participants. Telemed J Ehealth.

[56] Zheng Y, Terry MA, Danford CA, Ewing LJ, Sereika SM, Goode RW, Mori A, Burke LE (2018). Experiences of daily weighing among successful weight loss individuals during a 12-month weight loss study. West J Nurs Res.

[57] Leung AWY, Chan RSM, Sea MMM, Woo J. An overview of factors associated with adherence to lifestyle modification programs for weight management in adults. Int J Environ Res Public Health. 2017;14(8):922. 10.3390/ijerph14080922. 10.3390/ijerph14080922 PMC5580624 28813030

[58] Ufholz, K. Peer support groups for weight loss. Curr Cardiovasc Risk Rep. 2020;14(10):1-19. 10.1007/s12170-020-00654-4

[59] Michie S, Abraham C, Whittington C, McAteer J, Gupta S (2009). Effective techniques in healthy eating and physical activity interventions: a meta-regression. Health Psychol.

[60] Aguilar-Martínez A, Solé-Sedeño JM, Mancebo-Moreno G, Medina FX, Carreras-Collado R, Saigí-Rubió F (2014). Use of mobile phones as a tool for weight loss: a systematic review. J Telemed Telecare.

[61] Jensen MD, Ryan DH, Apovian CM, Ard JD, Comuzzie AG, Donato KA (2014). 2013 AHA/ACC/TOS guideline for the management of overweight and obesity in adults: a report of the American college of cardiology/American Heart Association task force on practice guidelines and the obesity society. Circulation.

[62] Butryn ML, Godfrey KM, Martinelli MK, Roberts SR, Forman EM, Zhang F (2019). Digital self-monitoring: does adherence or association with outcomes differ by self-monitoring target?. Obes Sci Pract.

[63] Belza B, Toobert D, Glasgow R. RE-AIM for Program Planning: Overview and Applications 2013. Available from: https://fromhungertohealth.files.wordpress.com/2013/02/re-aim_issue_brief.pdf.

